# Xenin is a novel anorexigen in goldfish (*Carassius auratus*)

**DOI:** 10.1371/journal.pone.0197817

**Published:** 2018-05-23

**Authors:** Brent Kerbel, Kimberly Badal, Lakshminarasimhan Sundarrajan, Ayelen Blanco, Suraj Unniappan

**Affiliations:** 1 Department of Biology, York University, Toronto, Ontario, Canada; 2 Laboratory of Integrative Neuroendocrinology, Department of Veterinary Biomedical Sciences, Western College of Veterinary Medicine, University of Saskatchewan, Saskatoon, Saskatchewan, Canada; Universidade de Vigo, SPAIN

## Abstract

Xenin, a highly conserved 25 amino acid peptide cleaved from the N-terminus of the coatomer protein alpha (COPA), is emerging as a food intake regulator in mammals and birds. To date, no research has been conducted on xenin biology in fish. This study aims to identify the *copa* mRNA encoding xenin in goldfish (*Carassius auratus*) as a model, to elucidate its regulation by feeding, and to describe the role of xenin on appetite. First, a partial sequence of *copa* cDNA, a region encoding xenin, was identified from goldfish brain. This sequence is highly conserved among both vertebrates and invertebrates. RT-qPCR revealed that *copa* mRNAs are widely distributed in goldfish tissues, with the highest levels detected in the brain, gill, pituitary and J-loop. Immunohistochemistry confirmed also the presence of COPA peptide in the hypothalamus and enteroendocrine cells on the J-loop mucosa. In line with its anorexigenic effects, we found important periprandial fluctuations in *copa* mRNA expression in the hypothalamus, which were mainly characterized by a gradually decrease in *copa* mRNA levels as the feeding time was approached, and a gradual increase after feeding. Additionally, fasting differently modulated the expression of *copa* mRNA in a tissue-dependent manner. Peripheral and central injections of xenin reduce food intake in goldfish. This research provides the first report of xenin in fish, and shows that this peptide is a novel anorexigen in goldfish.

## Introduction

Xenin is a 25 amino acid peptide cleaved from the N-terminus of its precursor coatomer protein-α (COPA) [1], by an aspartic protease, presumably cathepsin E [2,3]. It was first described in the human gastric mucosa [1], and later observed as well in endocrine cells of the duodenum and jejunum of primates and dogs, where it colocalizes with a subpopulation of gastric inhibitory polypeptide (GIP)-immunoreactive cells [4]. Although the gastrointestinal system shows the highest levels of xenin expression in both humans and dogs [5], it has been detected in the peripheral circulation of humans [1,6], and in the hypothalamus, lung, pancreas, liver, heart, kidney, adrenal gland, testis, and skin of dogs [5].

The observation that xenin is expressed in tissues that regulate food intake suggests that it might be involved in such a process. Indeed, several reports have provided clear evidence for the inhibitory role of xenin on food intake in mammals and birds. Acute administration (both central and peripheral) of xenin reduces food intake in mice [7–10], rats [7,11], and chicks [12,13]. Leckstrom and coworkers [8] found that intraperitoneal (IP) injection of xenin stimulates hypothalamic neurons directly involved in the regulation of food intake, including neurons in the paraventricular nucleus, arcuate nucleus (ARC) and dorsomedial nucleus of the hypothalamus, and the lateral hypothalamic area of mice, suggesting that xenin-induced reduction of food intake is mediated by central signaling in those nuclei. Further studies reported that xenin-induced satiety is partially mediated through a CRF-dependent [7], but not leptin- or melanocortin-dependent [8] signaling pathway. Besides acting at a central level, it has been reported that xenin reduces/delays gastric emptying in mice [14], chicks [12] and humans [15], thus suggesting that it might also induce satiety by acting directly on the gastrointestinal tract. In addition to reducing food intake, xenin has been reported to augment duodenal anion secretion in rats [16], to reduce body weight gain in mice [9], to cause alterations in lipid metabolism in mice adipose tissue [10] and to regulate blood glucose levels in mice and rats [17–19], among other functions. Therefore, xenin is emerging as a multifunctional peptide with predominant anorectic effects in mammals and birds.

To the best of our knowledge, no information is available on xenin in non-mammals other than birds, except for the mRNA sequences of *copa* predicted for some species and made available in the GenBank. This study aims to investigate whether xenin is present in goldfish (*Carassius auratus*), a well-characterized representative model in non-mammalian neuroendocrinology. The objectives of this project were to: (i) characterize goldfish *copa* cDNA, (ii) observe the expression pattern of *copa* in goldfish tissues, (iii) examine both periprandial and food deprivation-induced changes in *copa* mRNA expression in goldfish, and (iv) observe the effects of IP and intracerebroventricular (ICV) administration of xenin on goldfish food intake.

## Materials and methods

### Animals

Common goldfish (*Carassius auratus*), 30–40 g, were purchased from a local distributor and maintained under a simulated natural photoperiod (14 h light: 10 h dark) in temperature controlled (19 °C ± 1 °C) water. Fish were daily fed a commercial pellet diet (Martin Profishent, Ontario, Canada). Fish were anesthetized with 0.15% tricainemethane sulfonate (TMS; Syndel Laboratories, British Columbia, Canada) before any manipulation. All experimental protocols strictly adhered to the policies of the Canadian Council for Animal Care, and were approved by the York University Animal Care Committee.

### Peptide

Human xenin (Catalog #046–74; MLTKFETKSARVKGLSFHPKRPWIL) was purchased from Phoenix Pharmaceuticals (Burlingame, USA) with purity ≥95%. The molecular weight and purity of the peptide were confirmed by Electrospray Ionization Mass Spectroscopy and MALDI-TOF, and high performance liquid chromatography. Freshly made fish physiological saline [20] was used to reconstitute a new aliquot of the peptide on the day of the study.

### Xenin cDNA sequencing

*In silico* tools were used to find COPA gene/mRNA and protein sequencing information for *Micromonas sp*. (algae; XM_002501848.1), *Thalassiosira pseudonana* (diatom; XM_002291058.1), *Ciona intestinalis* (sea squirt; XM_002131228.1), *Saccoglos suskowalevskii* (acorn worm; XM_002731243.1), *Bombyx mori* (silkworm; NM_001172721.1), *Salmo salar* (salmon; NM_001140353.1), *Danio rerio* (zebrafish; NM_001001941.2), *Xenopus laevis* (African clawed frog; NM_001093019.1), *Xenopus tropicalis* (Western clawed frog; NM_001127994.1), *Meleagris gallopavo* (wild turkey; XM_003213951.1), *Gallus gallus* (chicken; NM_001031405.1), *Monodelphis domestica* (grey short-tailed opossum; XM_001369587.2), *Cricetulus griseus* (Chinese hamster; XM_003500279.1), *Cavia porcellus* (guinea pig; XM_003466569.1), *Rattus norvegicus* (rat; NM_001134540.1), *Mus musculus* (mouse; NM_009938.4), *Loxodonta Africana* (African bush elephant; XM_003415185.1), *Sus scrofa* (pig; XM_001928707.3), *Bos Taurus* (bull; NM_001105645.1), *Equus caballus* (horse; XM_001914729.2), *Nomascus leucogenys* (white-cheeked gibbon; XM_003259105.1), *Macaca mulatta* (rhesus macaque; XM_002801877.1), *Pan troglodytes* (common chimpanzee; XM_001171563.2), and *Homo sapiens* (human; NM_001098398.1), from GenBank (http://www.ncbi.nlm.nih.gov/genbank/). With the exception of zebrafish and salmon, no other teleost COPA sequences have been annotated.

Total RNA was extracted from goldfish brain using the TRIzol RNA isolation reagent (Invitrogen, Ontario, Canada) and checked for purity by optical density (OD) absorption ratio (OD 260 nm/OD 280 nm) using a Nanodrop 2000 (Thermo Scientific, Ontario, USA). Synthesis of cDNA was carried out using iScript cDNA synthesis kit (BioRad, Ontario, Canada) following manufacturer’s instructions. The cDNAs were used as templates for reverse transcription-PCR using primers based on the zebrafish *copa* mRNA sequence available on GenBank (NM_001001941.2) (Table 1). The subsequent partial amplification (~300 bp) of goldfish *copa* cDNA was gel extracted and purified using a QIAquick Spin kit (QIAGEN, Ontario, Canada). DNA sequencing was performed using the Applied Biosystems DNA Sequencer (3130xL) using the BigDyeH Terminator chemistry at the York University Core Molecular Biology and DNA Sequencing Facility. The sequence obtained was 274 bp length and showed similarity to the N-terminus of known COPA sequences in a BLAST search (http://blast.ncbi.nlm.nih.gov/Blast.cgi).

**Table 1:** Sequences of forward and reverse primers used in this study.

Gene	Sequence (5’ to 3’)	Amplicon size (bp)
*Copa*	F: CGCAAAGTCCCTTCCTACCGG R: CCTCCAGACACAAACAGAGGC	317
*β-actin*	F: CTACTGGTATTGTGATGGACTCCG R: TCCAGACAGAGTATTTGCGCTCAG	579

F, Forward; R, Reverse

Goldfish partial COPA nucleotide sequence obtained was converted into amino acid sequence using ExPASy Translate freeware (http://web.expasy.org/translate/). Using ClustalW2 general purpose multiple sequence alignment program for DNA and protein (http://www.ebi.ac.uk/Tools/msa/clustalw2/), the nucleotide and translated partial COPA sequence and the xenin region (first 25 aa of the translated partial COPA sequence) were aligned with sequences from all the aforementioned species obtained from GenBank.

### Tissue distribution of *copa* mRNAs in goldfish

Six goldfish (three female and three male) were anesthetised and euthanized via spinal transection. The following tissues were collected: whole brain (whole brain tissue, except the hypothalamus), hypothalamus, pituitary, eye, gill, heart, anterior intestine (J-loop), midgut, posterior intestine (rectum), gall bladder, kidney, liver, ovary, testes, and skin. Total RNA was extracted and cDNA was synthesized as described above. Real-time quantitative PCR (RT-qPCR) was carried out using iQ^™^ SYBR^®^ Green Supermix (Bio-Rad, Ontario, Canada) on a CFX96 Real-Time PCR Detection System (Bio-Rad, Ontario, Canada). Primers used for target gene *copa* and reference gene *β-actin* are shown in Table 1. RT-qPCR conditions consisted of an initial denaturation step at 95 °C for 3 min, and 35 cycles of 95 °C for 30 sec, 50 °C (for *copa*) or 59°C (for *β-actin*) for 30 sec and 73°C for 30 sec. Purity of each amplicon was confirmed by a melting curve, which consisted of a slow ramp from 55 °C to 95 °C with SYBR Green readings taken every 1 °C for 35 cycles. Additionally, the template products were electrophoresed to confirm the amplicon size (~300 bp) and validated by DNA sequencing as described above. Data was analyzed by CFX Manager^™^ software, version 2.1. The 2-ΔΔCt method [21] was used to determine the relative mRNA expression.

### Xenin-like immunoreactivity

Whole brain and J-loop samples from goldfish were collected, immediately transferred to 4% paraformaldehyde and incubated overnight (~12 h) at 4 °C. Subsequently, the forebrain and J-loop tissues were washed with 70% ethanol, embedded in paraffin wax and sagitally (brain) or longitudinally (J-loop) sectioned (5 μm thick). Sections were deparaffinized with xylene, rehydrated in a graded ethanol series and quenched for 30 minutes with 3% hydrogen peroxide. Sections were then incubated with rabbit anti-alpha COP 1 primary antibody (Abcam, Cambridge, USA; 1:300 dilution) for 12 h at room temperature. Slides were then washed and incubated with goat anti-rabbit Texas Red^®^ IgG (Vector Laboratories, Burlington, Canada; 1:100 dilution) for 1 h at 37 °C. A separate set of negative control slides were only treated with the secondary antibody. Additionally, primary antibody pre-absorbed in human xenin (1:10 molar ratio) overnight was used as pre-absorption control for confirming the antibody specificity. Primary and secondary antibodies were diluted in Dako Cytomation^®^ antibody diluent (Dako, Burlington, Canada). Lastly, after washing, slides were mounted with Vectashield^®^ mounting medium containing DAPI (Vector Laboratories). Sections were viewed using a Nikon Eclipse Ti-inverted fluorescence microscope (Nikon, Mississauga, Canada) connected to a Dell HP Workstation computer and NIS-elements basic research imaging software (Nikon, Mississauga, Canada).

### Periprandial profile of *copa* mRNAs

Seven groups of weight-matched goldfish (n = 4/group) were acclimated for 14 days to tank conditions and fed daily at a scheduled time (12:00, 0 h) during 14 days. Hypothalamus, J-loop, and liver samples were collected at 3 h prior to feeding, 1 h prior to feeding, upon commencement of feeding (0 h), 1 h after feeding (+1 h fed) and 3 h after feeding (+3 h fed). Two unfed groups were sampled at +1 h, and +3 h and served as the unfed group (+1 h unfed, +3 h unfed). Total RNA extraction, cDNA synthesis, and quantification of *copa* mRNA expression by RT-qPCR were conducted as described above.

### Food deprivation study

Five groups of weight-matched goldfish (n = 6 fish/group) were acclimated to tank conditions and fed daily at a scheduled time (11:00 am) for 14 days. Two groups of fish were then not exposed to food for 3 or 7 days, while the two control groups were fed daily. Hypothalamus, J-loop, and liver were sampled on days 3 and 7 from the fasted fish and from the fed fish at 1 h post-scheduled feeding time. After a 6-day fasting, one additional unfed group was re-fed on the 7th day and sampled at 1 h post-re-feeding. Total RNA extraction, cDNA synthesis, and RT-qPCR were conducted as described above.

### Effects of xenin on food intake

Goldfish were acclimated during 12 days to experimental tanks, each housing two weighed-matched fish. During acclimation, fish were fed a daily ration of 4% body weight (BW) of pellets per fish at 11:00 am. The day of the experiment, fish were injected with saline or xenin either intraperitoneally or intracerebroventricularly. IP injections were conducted as follows. Fish were anesthetized and injected with a 100 μl volume of fish saline or xenin (1, 10, and 100 ng/g BW) dissolved in fish saline using a 1 ml syringe and 25G needle (BD, Oakville, Canada) into the peritoneal cavity. Subsequently, fish were placed back in their respective tanks and allowed 5 min to recover. ICV injections were conducted as follows. Fish were anesthetized, weighed, wrapped in a moistened Kimwipe^®^ (KIMTECH Science^™^), and then positioned in a wet sponge with centre cut down to form a trough, allowing the fish to be held securely and so that the dorsal side of the fish was facing up. Forceps and a dentist drill equipped with a circular saw were used to open a dorsal flap on the frontal cartilage to expose the brain. The fish were then securely held on a stereotaxic apparatus (World Precision Instruments, Sarasota, FL) and injected with a 2 μl volume of fish saline or xenin (0.1, 1, or 10 ng/g BW) dissolved in fish saline using a 10 μl Hamilton microsyringe (Hamilton Company, Reno, NV). The Hamilton microsyringe was stereotaxically positioned in the third ventricle of the fish brain using the coordinates previously described by Peter and Gill (1975). After injecting and suturing, the fish were placed back into their respective tanks and allowed 5 min to recover. After recovery, all fish were fed a ration of food equal to 6% BW per fish. Uneaten pellets were collected after 1 h, dried for 24 h in an oven, and food intake of peptide treated fish was compared to that of the controls.

### Statistical analysis

Statistical differences in the amount of mRNA transcripts and food intake were assessed using the Student *t*-test (for the 3-day fasting experiment), and one-way ANOVA followed by Student-Newman-Keuls multiple comparison (for the rest of studies), after data were checked for variances homogeneity. Significance was assigned when p<0.05. All analyses were carried out using GraphPad Prism 4 (San Diego, USA).

## Results

### A partial sequence of *copa* mRNA was identified in goldfish

**Fig 1:**
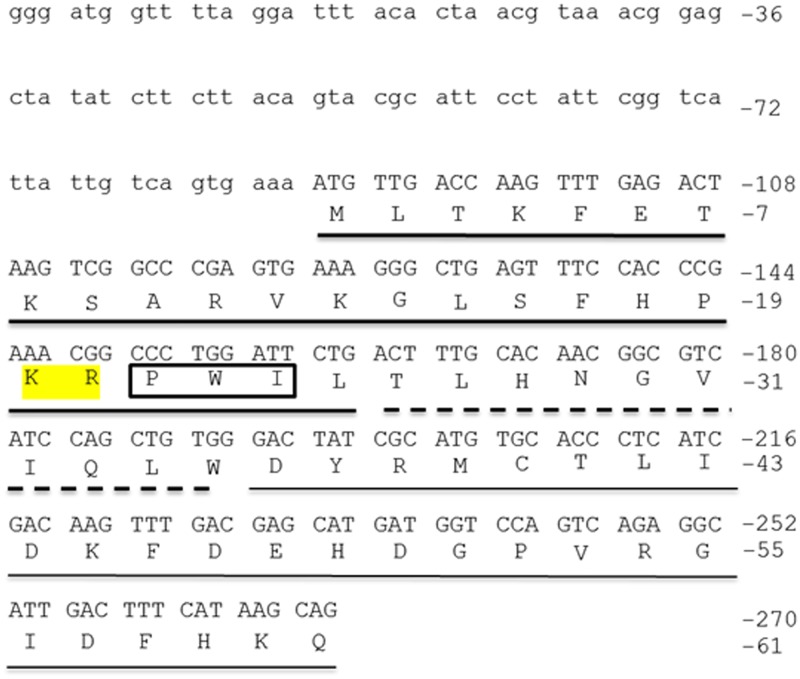
Nucleotide and amino acid sequence of the N-terminus of goldfish COPA. The lower case letters indicate the 5’-untranslated region (UTR) of goldfish COPA. The start codon (Met, M) is found at nucleotides 88–90. The thick underlined region indicates goldfish xenin. The broken-underlined region indicates the proxenin sequence. The thin underlined region indicates the remaining aa of the partial COPA sequence obtained. The highlighted aa are required for xenin receptor-binding. The 3 aa in the box are the putative cathepsin E recognition site. NCBI Accession Number: JQ929912.

**Fig 2:**
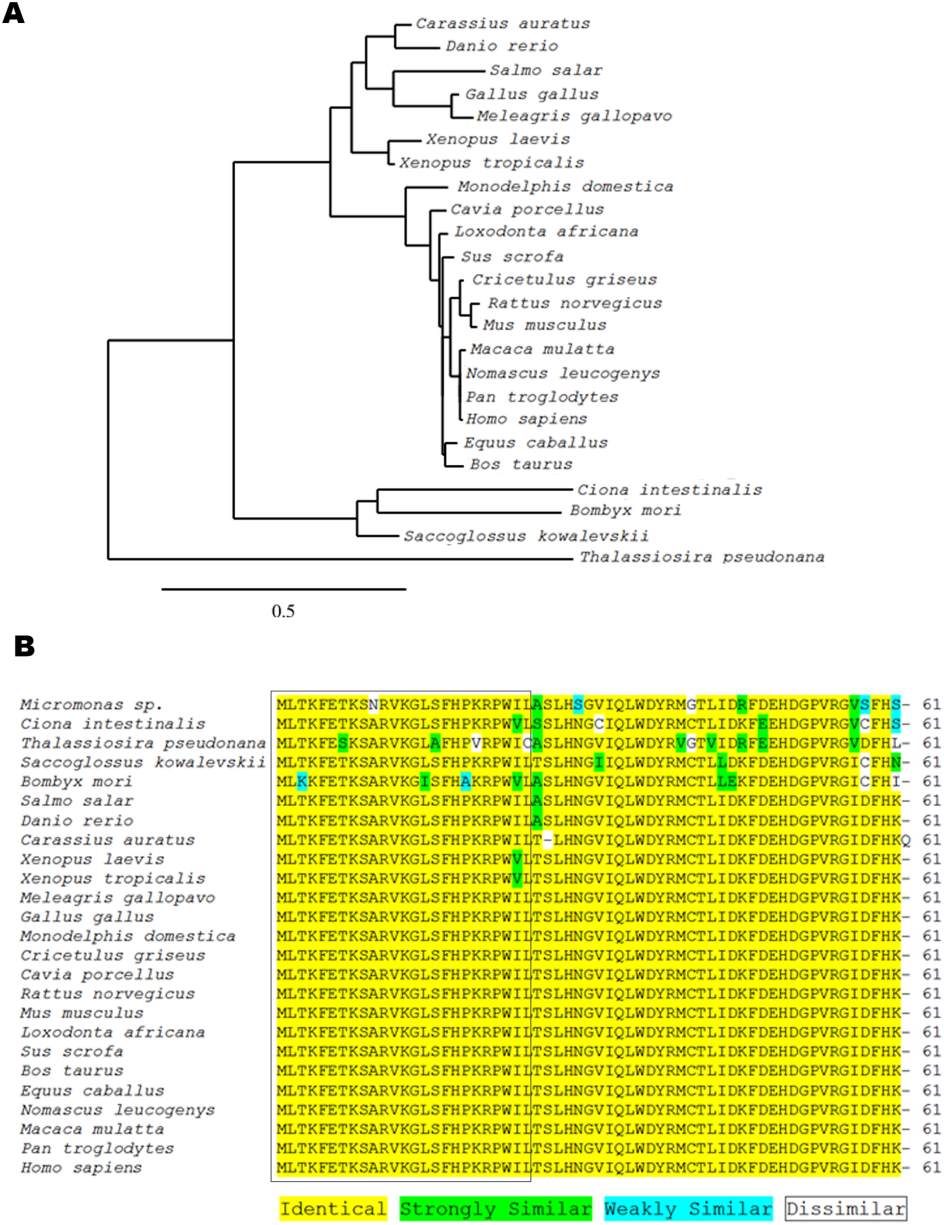
Phylogenetic analysis of the partial COPA sequence obtained from goldfish. **(A)** Phylogenetic tree showing the evolutionary relationships of the obtained nucleotide sequence of goldfish *copa* with those of other species. Tree was inferred by the neighbor-joining method using the online tool www.phylogeny.fr. The scale bar indicates the average number of substitutions per position (a relative measure of evolutionary distance). The names of the species used for the alignment are provided in the figure. GenBank accession numbers of the sequences used are as follows: *Bombyx mori*, NM_001172721.1; *Bos taurus*, NM_001105645.1; *Carassius auratus*, JQ929912.1; *Cavia porcellus*, XM_003466569.3; *Ciona intestinalis*, XM_002131228.4; *Cricetulus griseus*, XM_007629307.2; *Danio rerio*, NM_001001941.2; *Equus caballus*, XM_023640888.1; *Gallus gallus*, NM_001031405.2; *Homo sapiens*, NM_001098398.1; *Loxodonta africana*, XM_023554162.1; *Macaca mulatta*, XM_015113467.1; *Meleagris gallopavo*, XM_003213951.3; *Monodelphis domestica*, XM_016430274.1; *Mus musculus*, NM_009938.4; *Nomascus leucogenys*, XM_012510889.1; *Pan troglodytes*, XM_001171563.4; *Rattus norvegicus*, NM_001134540.1; *Saccoglossus kowalevskii*, XM_002731243.2; *Salmo salar*, XM_014140665.1; *Sus scrofa*, XM_001928697.6; *Thalassiosira pseudonana*, XM_002291058.1; *Xenopus laevis*, NM_001093019.2; *Xenopus tropicalis*, NM_001127994.1. **(B)** Alignment of the first 61 aa of COPA from an algal species, invertebrate species, teleost fishes, amphibians, avians, and mammals. The species names are provided on the left-hand side of the alignment and the number of aa is present on the right-hand side of the alignment. The coloured aa highlights the differences in conservation between species. The first 25 aa (which correspond to the xenin region) are boxed.

A partial sequence (272 bp) of *copa* was obtained from goldfish brain cDNA after RT-PCR using zebrafish *copa* primers (Fig 1). The NCBI basic local alignment search tool (BLAST; http://blast.ncbi.nlm.nih.gov/Blast.cgi) confirmed this amplicon to be highly similar to zebrafish *copa*. Using ClustalW2, *copa* mRNA sequences from zebrafish, African clawed frog, chicken, rat, and mouse were aligned with the newly obtained goldfish sequence (S1 Fig). The goldfish sequence was found to be highly similar to zebrafish *copa* (85% similar). Also, it was 66% similar to mouse, 62% similar to rat, 61% similar to chicken, and 60% similar to frog *copa* sequences. The translation of the goldfish *copa* partial sequence into protein sequence revealed a 61 aa long partial peptide sequence (Fig 1). The phylogenetic analysis performed shows that goldfish *copa* mRNA sequence appears closely related to other cyprinids (zebrafish), in agreement with the concept of traditional taxonomy (Fig 2A). This sequence was extremely similar to other known sequences of COPA, including both invertebrates and vertebrates. Indeed, the xenin region of goldfish COPA (first 25 aa) was observed to be identical to acorn worm, salmon, wild turkey, Chinese hamster, mouse, bull, rhesus macaque, human, chimpanzee, grey short-tailed opossum, rat, pig, white-cheeked gibbon, zebrafish, chicken, guinea pig, African elephant, and horse xenin, 96% similar to African clawed frog, Western clawed frog, sea squirt, and algae xenin, and 88% similar to diatom and silkworm xenin sequences (Fig 2B). The proposed cathepsin E cleavage site, P-X-X, where X represents a hydrophobic aa, is found at aa 22–24 in the goldfish sequence (Fig 2). In addition, the Lys-Arg sequence reported as being crucial for xenin-receptor binding is conserved in all xenin sequences except in the diatom (Fig 2).

### *Copa* mRNAs are widely distributed in goldfish tissues

**Fig 3:**
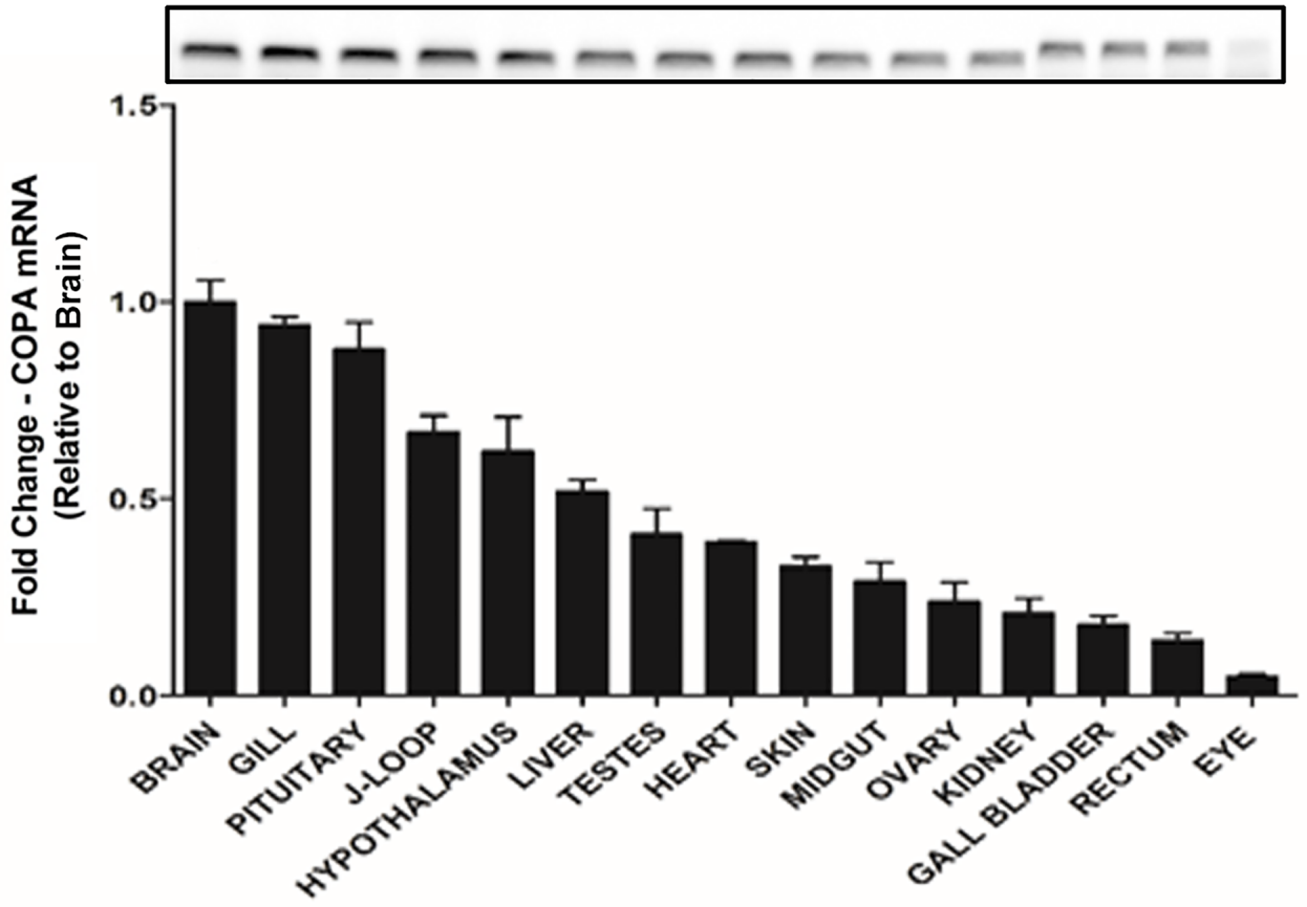
Differential expression of *copa* mRNA in goldfish tissues. Real-time quantitative PCR (RT-qPCR) was used to quantify the level of *copa* mRNA expression in each tissue. The mRNA expression detected was normalized to *β-actin* and represented relative to the brain (tissue with the highest expression). Data are presented as mean + SEM (n = 6 fish). Agarose gel image of the PCR products is shown at the top.

Expression of *copa* transcripts was detected in all goldfish tissues tested (Fig 3). The highest *copa* mRNA expression was observed in the whole brain, followed by the gill, pituitary, J-loop and hypothalamus. Moderate mRNA levels were found in heart, other intestinal regions, liver, kidney and gonads. Expression of *copa* was found to be minimal in eye.

### The goldfish hypothalamus and J-loop contain cells immunopositive to xenin

**Fig 4:**
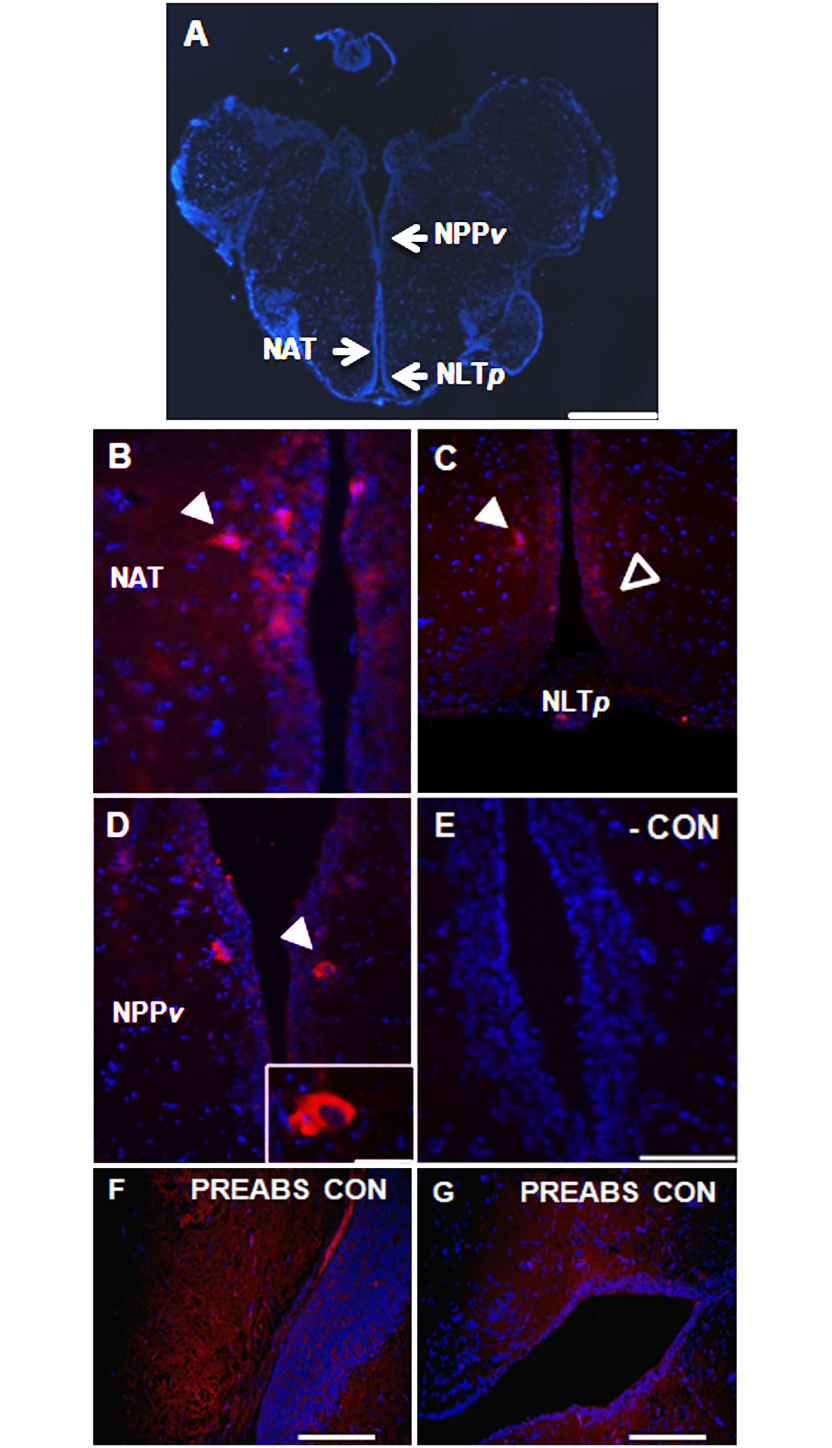
Xenin-like immunoreactivity in the goldfish brain. (**A**) Sagittal view of a goldfish brain stained with DAPI (blue). Arrows indicate the region of the posterior periventricular nucleus (NPP*v*), nucleus anterior tuberis (NAT), and posterior nucleus lateralis tuberis (NLT*p*). Scale bar = 500 μm. (**B–D**) Immunohistochemical staining of goldfish NAT, NLT*p* and NPP*v* neurons for xenin-like immunoreactivity (red). All images are merged with DAPI showing nuclei in blue. Arrows point to immunopositive cells. In C, open arrows point to xenin-like immunoreactive neurons along the ventricle, and closed arrows to positive neurons lateral to the ventricle. Scale bars = 50 μm (B), 100 μm (C, D), 20 μm (inset in D). (**E**) Image of a negative control slide stained with secondary antibody alone. Scale bar = 50 μm. (**F, G**) Transversal representative sections of goldfish brain treated with specific primary anti-xenin antibody pre-absorbed in xenin. Scale bars = 200 μm.

A sagittal view of the goldfish brain stained with anti-xenin antibody revealed presence of xenin-like immunoreactive cells within the goldfish hypothalamus (Fig 4A). A relatively high proportion of these cells were found in the nucleus anterior tuberis (NAT; Fig 4B) and the posterior nucleus lateralis tuberis (NLT*p*; Fig 4C). Scattered neurons immunopositive for xenin-like were also observed in the posterior periventricular nucleus (NPP*v*; Fig 4D). In all areas, xenin-like immunoreactivity was observed in the perikarya of neurons as well as the neuronal processes. No staining was observed in brain sections where primary antibody was either omitted (Fig 4E) or pre-absorbed with xenin (Fig 4F and 4G).

**Fig 5:**
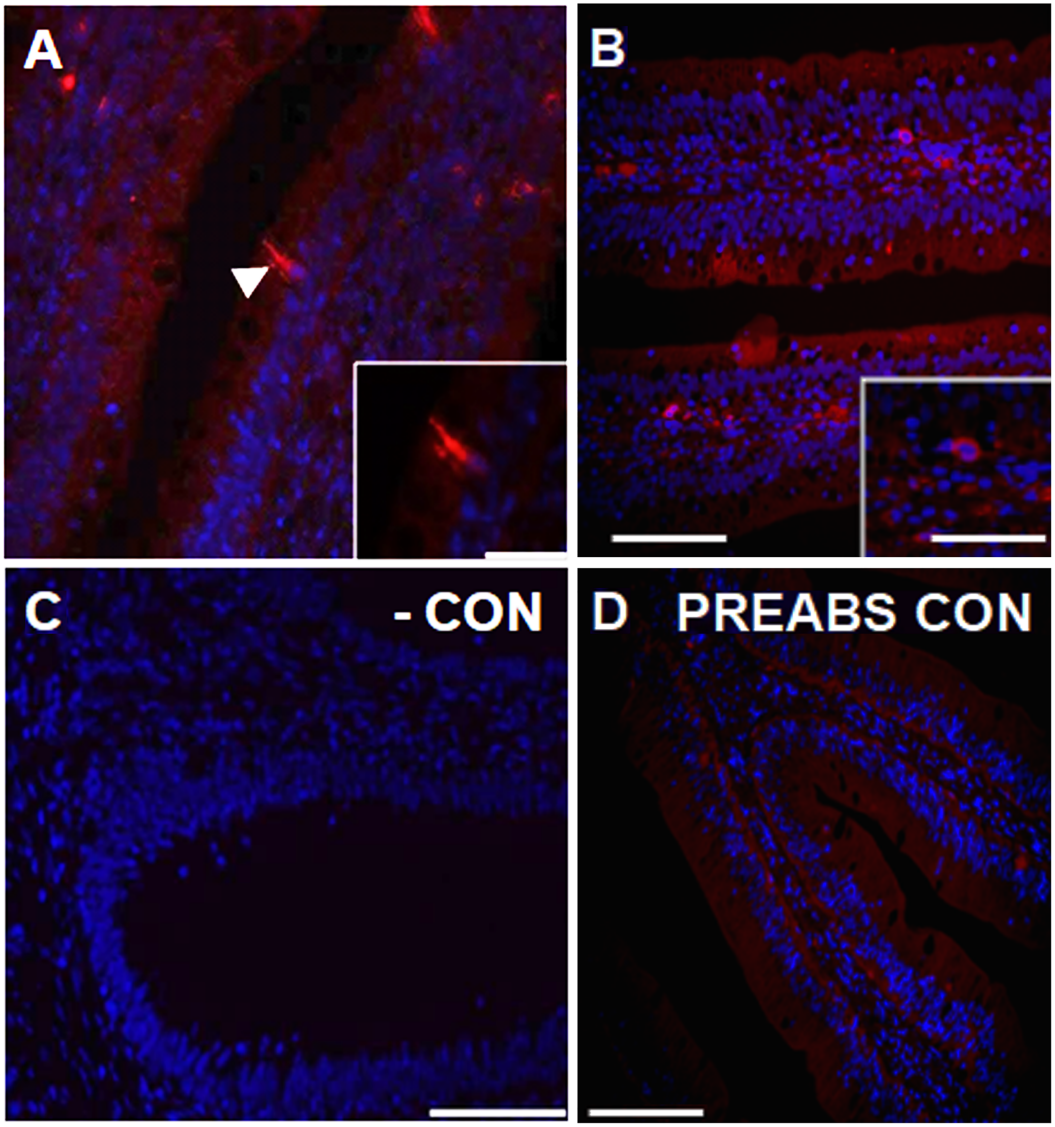
Xenin-like immunoreactivity in the goldfish J-loop (anterior intestine). (**A, B**) Representative sections of the goldfish J-loop stained for xenin-like immunoreactivity (red) and DAPI for nuclei (blue). Arrow points to an immunopositive cell. Scale bars = 100 μm (A), 100 μm (B), 20 μm (inset in A), 0.5 μm (inset in B). (**C**) Image of a negative control slide stained with secondary antibody alone. Scale bar = 100 μm. (**D**) Transversal representative section of goldfish J-loop treated with specific primary anti-xenin antibody pre-absorbed in xenin. Scale bars = 200 μm.

Immunostaining of goldfish J-loop revealed also presence of xenin-like immunoreactivity within enteroendocrine cells of the mucosa, as it can be observed in Fig 5A. These cells were scattered within the folds between villi and in apical regions of the villi. Xenin-like immunoreactivity was found in the cell bodies and within the thin processes of the enteroendocrine cells that project towards the lumen of the J-loop. There were also cells positive for xenin-like in the goldfish submucosa (Fig 5B). No staining was observed neither in negative (Fig 5C) nor pre-absorption (Fig 5D) control slides.

### Expression of *copa* mRNAs displays periprandial fluctuations in the goldfish hypothalamus and liver

**Fig 6:**
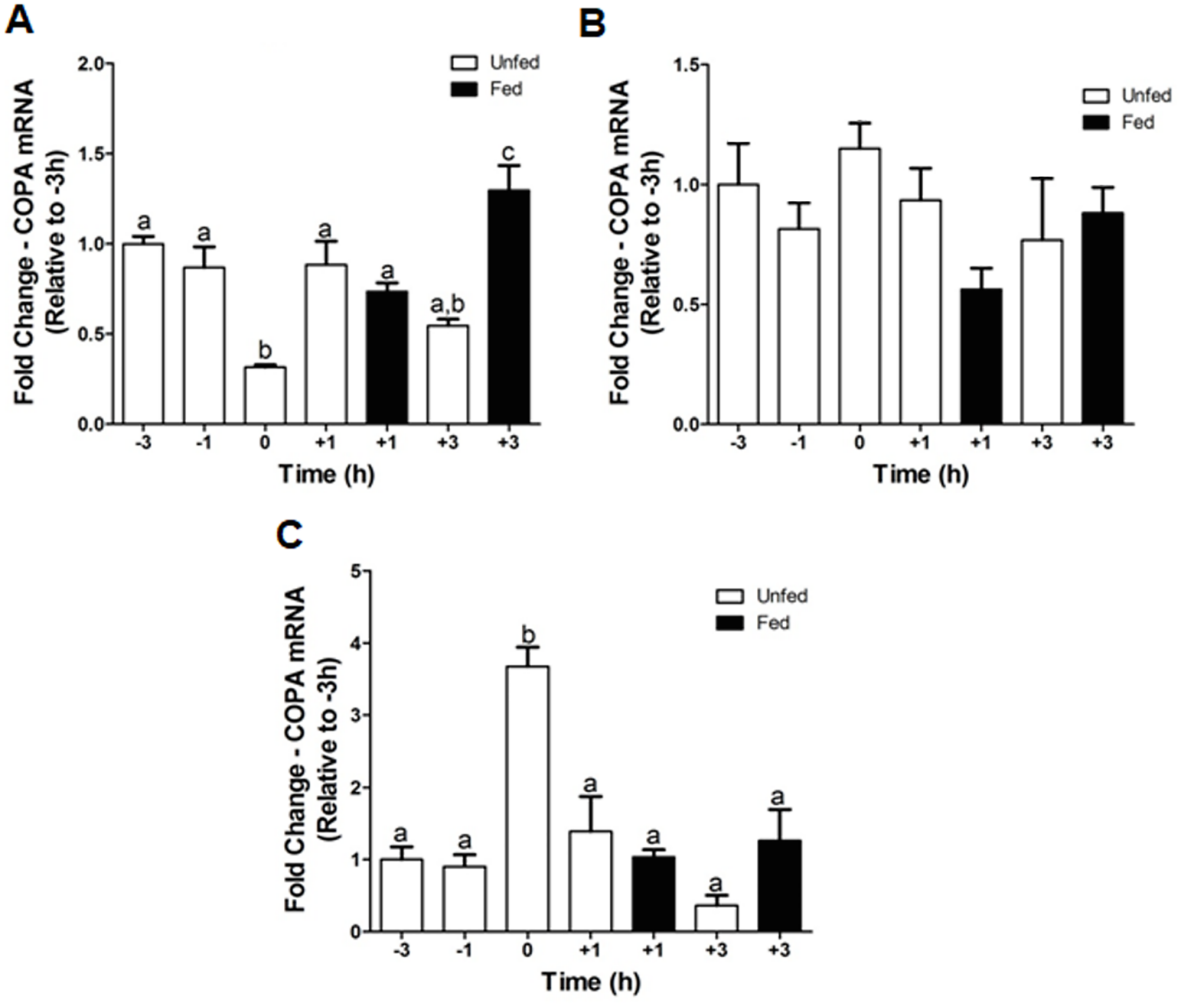
Pre- and post-prandial changes of *copa* mRNA expression in the goldfish hypothalamus (A), J-loop (B), and liver (C). The mRNA expression of *copa* was normalized to *β-actin* and represented relative to the -3 h scheduled feeding group. Data are presented as mean + SEM (n = 4 fish). Columns sharing a same letter are not statistically different (p < 0.05, one-way ANOVA and Student-Newman-Keuls tests).

Study of the periprandial expression profile of *copa* mRNAs revealed the existence of periprandial fluctuations in the goldfish hypothalamus (Fig 6A). These fluctuations were mainly characterized by a decrease in *copa* mRNA levels as the feeding time was approached, and an increase after feeding. Thus, expression levels of *copa* at feeding time (0 h) were ~68% and ~63% lower compared to its expression in fish sampled at 3 h (-3 h) and 1 h (-1 h) before feeding, respectively, and ~57% and ~75% lower compared to expression levels at 1 h (+1 h fed) and 3 h (+3 h fed) after feeding, respectively. Expression levels of *copa* were also upregulated at +1 h in those fish who missed the scheduled feeding compared to 0 h, but not at +3 h. Within the liver, *copa* mRNA expression at 0 h was significantly greater compared to all other groups (Fig 6C). The percent change in expression ranged from ~62% between 0 h and +1 h unfed to ~90% between 0 h and +3 h unfed. Levels of *copa* mRNAs were unaltered periprandially in the goldfish J-loop (Fig 6B).

### Fasting modulates the mRNA expression of *copa* in goldfish tissues

**Fig 7:**
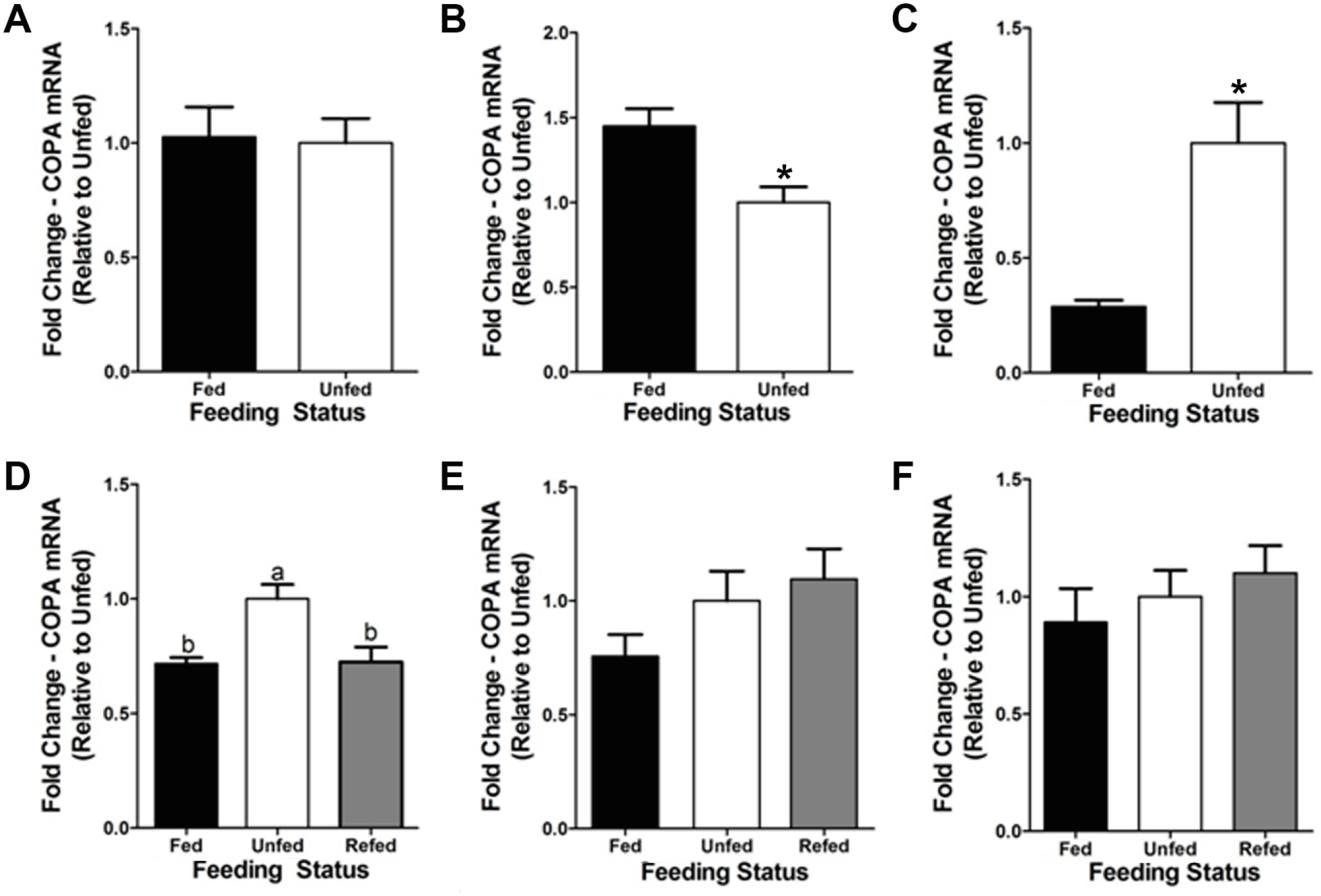
Fasting-induced changes of *copa* mRNA expression in goldfish central and peripheral tissues. (**A–C**) Expression of *copa* mRNAs after a 3-day fasting period in the goldfish hypothalamus (A), J-loop (C), and liver (E). The mRNA expression of *copa* was normalized to *β-actin* and represented relative to the unfed group. Data are presented as mean + SEM (n = 4 fish). Columns with a different letter are statistically different (p < 0.05, t-test). (**D–F**) Expression of *copa* mRNAs after a 7-day fasting period and refeeding in the goldfish hypothalamus (D), J-loop (E), and liver (F). The mRNA expression of *copa* was normalized to *β-actin* and represented relative to the unfed group. Data are presented as mean + SEM (n = 4 fish). Columns with a different letter are statistically different (p < 0.05, one-way ANOVA and Student-Newman-Keuls tests).

Fasting during 3 days was observed to significantly downregulate (~45%) mRNAs encoding *copa* in the goldfish J-loop (Fig 7B) and to cause a significant increase in their expression (~70%) in the liver (Fig 7C). Expression of *copa* was unaltered in the hypothalamus of fish fasted for 3 days (Fig 7A). At a longer time, we observed that hypothalamic mRNA expression of *copa* was elevated in fish fasted for 7 days (~30%) compared to daily fed fish. Refeeding of fasted fish the day of the experiment reverted the fasting-induced upregulation of *copa* mRNAs (Fig 7D). There were no significant changes in mRNA levels of *copa* in the J-loop and liver of goldfish food-deprived during 7 days compared to control fed fish (Fig 7E and 7F).

### Xenin reduces food intake in goldfish

**Fig 8:**
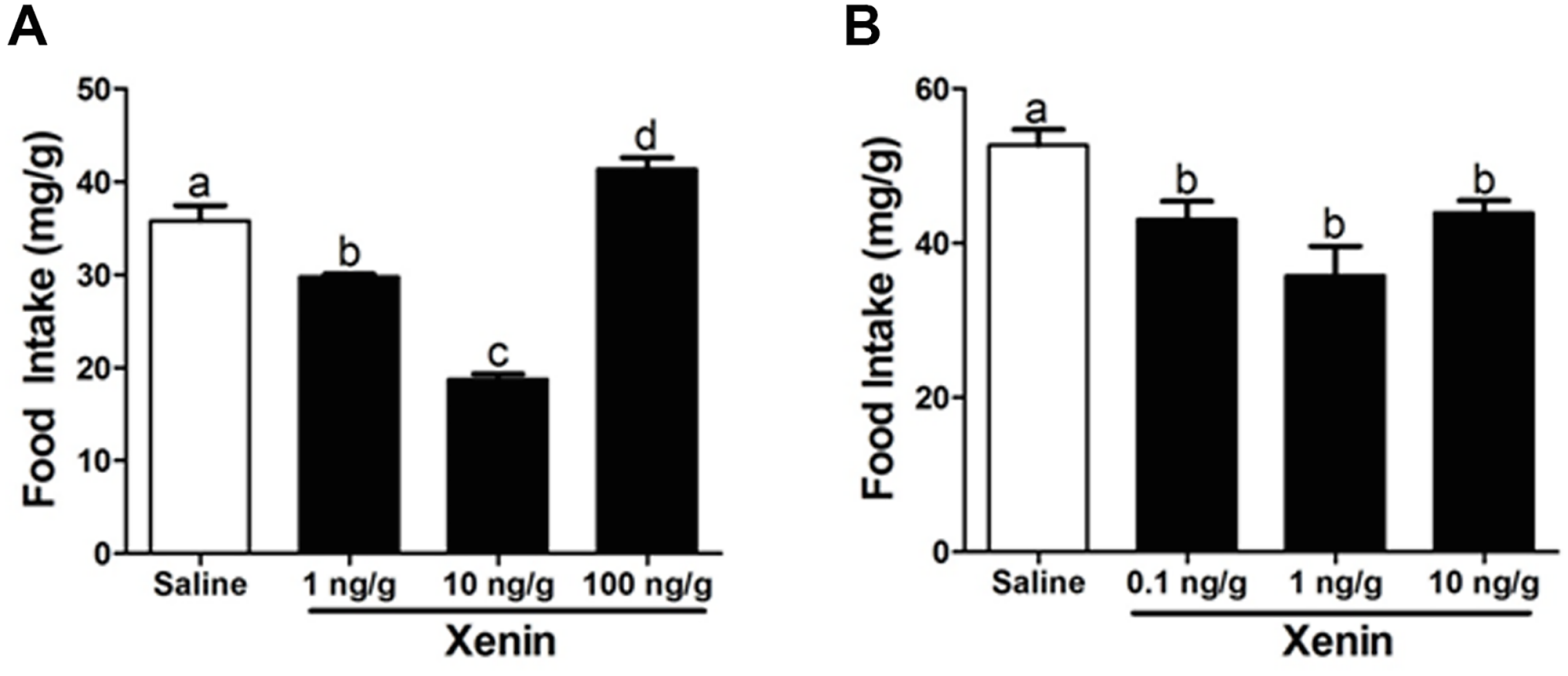
Effects of IP (A) and ICV (B) administration of xenin on food intake. Data are presented as mean + SEM (n = 6 fish for IP, 4 fish for ICV). Columns with a different letter are statistically different (p < 0.05, one-way ANOVA and Student-Newman-Keuls tests).

A single IP injection of 1 ng/g and 10 ng/g BW xenin was observed to reduce goldfish food intake by ~17% and ~48% (respectively) when compared to saline treated control fish. The highest dose tested, 100 ng/g BW, resulted in a ~13% increase in goldfish food intake (Fig 8A). ICV administration of all xenin doses tested (0.1, 1 and 10 ng/g) reduced food intake when compared to fish injected with saline. The magnitude of the inhibitory response on appetite was of ~18%, ~32% and ~17%, respectively (Fig 8B).

## Discussion

This study offers the first report of xenin in fish, a peptide that has been characterized as an anorexigen in mammals and birds. Our results showed that *copa* mRNA, distributed ubiquitously in goldfish tissues, encodes a very highly conserved xenin in goldfish. Goldfish xenin was found to be identical to all known mammalian, avian, and fish xenin sequences available in the GenBank. The high level of conservation of xenin throughout vertebrate evolution suggests that the biological functions of this peptide in mammals and birds may be present in fish as well. The goldfish xenin sequence obtained contains highly conserved motifs that are essential for the biological activity of xenin. The potential cathepsin E cleavage site [3], Pro-Trp-Ile, is also conserved at aa 22–24 at the C-terminus of goldfish xenin. This suggests that xenin can be processed by cathepsin E in goldfish. However, cathepsin E has yet to be characterized in fishes. Cathepsin D, a protease that shares similar substrate specificity with cathepsin E in mammals [22], has been characterized in zebrafish [23] and salmon [22]. Whether xenin is cleaved by cathepsin E, D, or another protease remains to be determined. Also, the dibasic Lys-Arg motif was conserved at aa 20–21 at the C-terminus of goldfish xenin. This motif is proposed to be important for xenin-receptor binding [24]. The proposed xenin receptor is neurotensin receptor 1 (NTR1; [25]. While the NTR1 is currently unknown in goldfish, using *in silico* techniques, Hwang and coworkers [26] found a predicted NTR in medaka, which is orthologous to the mammalian NTR1. Whether this NTR is present in goldfish and if xenin can bind to it to mediate its effects remain to be elucidated. There is a possibility that more than one xenin gene exists in goldfish due to the gene duplication. The zebrafish copa gene encodes 5 splice variants encoded in the same gene. Our sequencing confirms that all mRNAs we amplified from multiple tissues using the same primers were the same copa transcript. Further studies are required to determine whether additional genes encoding copa exist in goldfish.

Xenin was previously reported to be ubiquitously expressed in mammals. Using RIA, Hamscher and colleagues [5] found xenin in the hypothalamus, liver, heart, kidney, adrenal gland, pancreas, testes, and skin of dogs. Furthermore, they found xenin throughout the GI tract, including the gastric mucosa, duodenum, jejunum, ileum, and colon of humans and dogs [5]. The current study extends these findings by describing the tissue distribution of xenin in goldfish. Expression of *copa* mRNAs was found in all goldfish tissues examined. Although preliminary in nature, our findings suggest that gill and pituitary are abundant sources of *copa*, and potentially xenin as well. This observation might suggest that both *copa* and/or xenin are involved in the regulation of gill epithelial physiology (eg. ion transport) and pituitary hormone secretion in goldfish. Important levels of *copa* were also found in the hypothalamus and the intestinal tract (J-loop, midgut, and rectum) of goldfish. In addition, immunohistochemical stainings show xenin-like immunoreactivity in the two mentioned areas.

The NLT*p*, NAT, NPP*v* are known as central food intake regulating sites in goldfish. Peptides that regulate food intake have been described in these brain regions. For instance, immunoreactivity of the anorexigen CCK was described in neurons of the NLT*p*, NAT, and NPP*v* in goldfish [27]. Furthermore, we described the immunoreactivity of the anorexigen nesfatin-1 in the goldfish NLT*p* and NAT [28]. Orexigens, such as neuropeptide Y [29], were described in neurons of the NLT*p* in goldfish. The presence of endogenous xenin in the NLT*p*, NAT, and NPP*v* suggests a potential role for this peptide in the regulation of energy balance, especially feeding. Furthermore, it might point out to an interaction with other food intake regulating peptides present in these brain regions. In addition, the NLT is considered the teleostean homologue of the mammalian ARC, a major central food intake regulating site in mammals [30]. Interestingly, IP administered xenin was found to activate ARC neurons in mice [8]. Similarly, it can be hypothesised that peripheral xenin might activate xenin immunoreactive neurons in the NLT*p* of fish to induce satiety. However, further research is required to validate this hypothesis.

Enteroendocrine cells within the J-loop are known to secrete peptides that regulate food intake. CCK-like [27], nesfatin-1-like [28], and ghrelin-like [31] immunoreactivity have all been described in the enteroendocrine cells of the goldfish J-loop. Again, the localization of xenin-like immunoreactivity in enteroendocrine cells suggests that xenin may regulate goldfish food intake and may do so by interacting with other food intake regulating peptides present in enteroendocrine cells of the J-loop. Interestingly, xenin and CCK have very similar biological effects in mammals. Both peptides reduce food intake [7,32], inhibit gastric emptying [14,33], enhance gall bladder contractility [34,35], and stimulate exocrine pancreatic secretion [1,36]. The similar localization pattern and biological activity of xenin and CCK suggest they might be functionally related, although further investigation needs to be performed.

Feeding status is known to regulate the secretion of xenin in mammals. The concentration of xenin in human plasma is increased post-prandially [1,6]. Feurle and coworkers [6] found that the anticipation of food intake also stimulates xenin secretion. Sham feeding, described as the smelling and tasting food without swallowing, increases plasma concentrations of xenin [6]. In the present study, we first report alterations in *copa* mRNA expression in response to nutritional status in a non-mammalian vertebrate species. Our results showed that hypothalamic *copa* mRNA expression is reduced at the scheduled feeding time (0 h) and increased 3 h post-prandially. Expression levels of other anorexigens, such as nesfatin-1, are increased in the hypothalamus 3 h post-prandially [28]. However, the reduction of *copa* mRNA at the scheduled feeding time is unique amongst the food intake regulating peptides. This result provides further evidence that the xenin system is activated in anticipation of food intake; fish were trained to eat at this time but did not eat on the day of the study. It is possible that the anorectic endogenous xenin is attenuated at the regular feeding time to promote feeding. Likewise, xenin is increased 3 h post-prandially to signal satiety. While the implications on feeding are possible, careful examination in future experiments is required to determine whether a circadian pattern of xenin expression exist in goldfish. In addition, the change in *copa* mRNA expression at the 0 h in the liver was opposite to that in the hypothalamus. Increasing the expression of a potential anorexigen at the time of feeding does not appear beneficial. Perhaps the altered expression of *copa* in the liver is a response to regulate an unknown biological function of COPA/xenin and not food intake. Surprisingly, *copa* mRNA expression in the J-loop was unaltered periprandially. The stomach and duodenum are thought to be the primary source of peripheral xenin in mammals due to the high expression of xenin throughout the GI tract [5]. The lack of change of *copa* mRNA expression in the goldfish J-loop suggests otherwise in fish. However, only measurements of mRNA levels were performed in this study. It would be interesting to see if the synthesis of xenin, its secretion, and/or xenin protein levels in the gut are altered in a meal responsive manner. In addition, xenin appears to be a fast acting peptide; circulating levels increase 15 min post-meal and return to baseline levels 30 min post-meal in humans [1]. Therefore, the sampling times of 1 h and 3 h utilized in this experiment may be too long. Further periprandial studies should be conducted with shorter sampling times to verify meal responsiveness of *copa* in the goldfish gut.

Fasting also altered *copa* mRNA expression in goldfish tissues. In the J-loop, 3 days of fasting reduced *copa* mRNAs, but no changes were observed after a 7-day food deprivation period. These results suggest that COPA/xenin is involved in the short-term adaptation to starvation in the J-loop. In addition, *copa* mRNA expression was increased in the hypothalamus post-7 days of fasting and increased in the liver post-3 days of fasting. Similar to what was discussed above, an increase in the expression of a potential anorexigen during fasting does not appear beneficial, and it is thus plausible that the fasting-induced altered expression of hypothalamic and liver *copa* was a response to regulate another unknown biological function of COPA/xenin in goldfish. Again, *copa* mRNA expression is not indicative of synthesis or release of xenin, and further studies are required to determine if fasting alters the secretion of xenin in goldfish. Nevertheless, the increase in hypothalamic *copa* mRNA expression post-prandially and the reduction of *copa* mRNAs in the J-loop of fasted fish suggest that endogenous xenin is involved in regulating goldfish food intake.

Results presented here provide the first functional evidence for the appetite regulatory effects of xenin in fish. ICV injections of 0.1, 1, and 10 ng/g BW reduced goldfish food intake, indicating that xenin has a central effect on goldfish food intake. In addition, IP injections of xenin, at a dose of 1 or 10 ng/g BW, reduced food intake in goldfish. Alternatively, the IP injection of 100 ng/g BW dose resulted in a significant increase in food intake. This result is likely due to the desensitization of xenin signaling resulting from the high dose of xenin administered [37]. It is possible that multiple receptors mediate the central and peripheral effects of xenin on feeding. The effect of xenin on food intake is probably strongly influenced by the tissue distribution, receptor affinity, and receptor regulatory mechanisms in target tissues. The reasons for the dual effects of xenin (anorexigenic at lower dose and orexigenic at a higher dose) could certainly include the differences in receptor-mediated mechanisms. The effect of centrally injected xenin on food intake was weaker than peripherally injected xenin, suggesting that xenin-induced satiety may primarily be a peripherally controlled response. In support of this, xenin is known to reduce gastric emptying in mammals [14] and birds [12], a process known to result in reduced food intake [38]. Therefore, xenin may reduce food intake by acting in the periphery to increase the transit time of food through the GI system. However, further research is required to determine the mechanism(s) of xenin-induced satiety in fish. In addition, studies to test both xenin doses lower and higher to what we studied are warranted to elucidate its full dose response effects on food intake.

In summary, the present study provides the first set of data characterizing xenin in fish. Our major finding were: (i) goldfish COPA is highly conserved among other vertebrates, (ii) COPA/xenin is present in important feeding-regulating areas, including the hypothalamus and gut, (iii) *copa* mRNA expression in the hypothalamus and J-loop are altered in response to feeding status and fasting, respectively, and (iv) exogenous xenin reduces feeding in goldfish. Taken together, these data provide strong support for the anorexigenic action of xenin in goldfish. Future studies will be focused on determining the mechanism of action of xenin in reducing food intake, and on investigating other putative roles of this peptide in fish.

## Supporting information

S1 Fig Alignment of the nucleotide sequence of COPA from goldfish and other vertebrate species. The species names are provided on the left-hand side of the alignment and the number of nucleotide is present on the right-hand side of the alignment. The coloured nucleotide highlights the primers used for obtaining the goldfish sequence.
(TIF)
