# Supplementary figures and images for: Xenin is a novel anorexigen in goldfish (Carassius auratus)
Source: PLoS One. 2018 May 23;13(5):e0197817. doi: 10.1371/journal.pone.0197817 (PMC5965858; doi:10.1371/journal.pone.0197817)

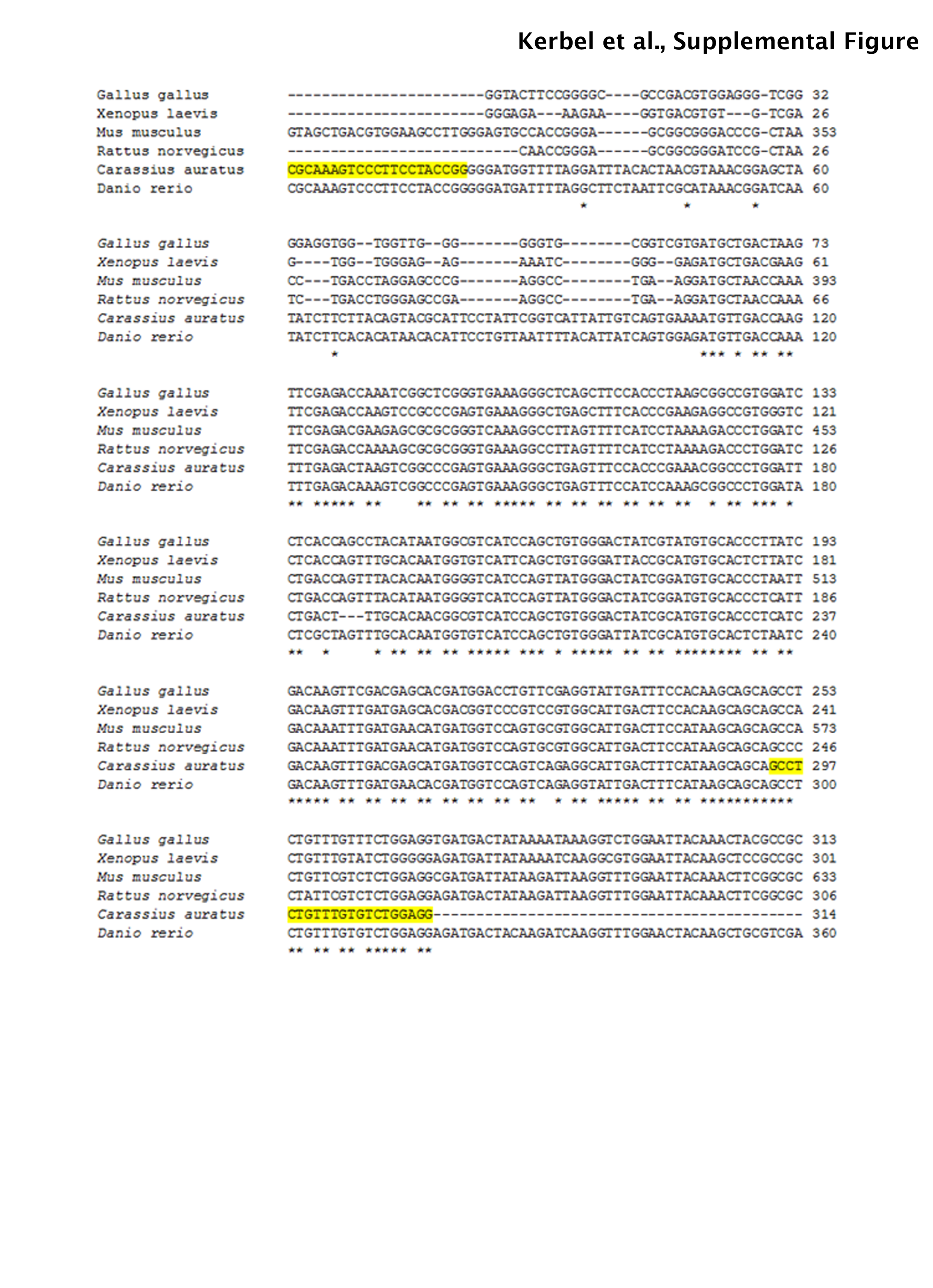

Supplement: S1 Fig — The species names are provided on the left-hand side of the alignment and the number of nucleotide is present on the right-hand side of the alignment. The coloured nucleotide highlights the primers used for obtaining the goldfish sequence. (TIF) [file pone.0197817.s001.tif]
